# What works to support carers of older people and older carers? an international evidence map of interventions and outcomes

**DOI:** 10.1186/s12877-024-04897-3

**Published:** 2024-03-29

**Authors:** Gemma Spiers, Michelle M.C. Tan, Jayne L Astbury, Alex Hall, Nisar Ahmed, Kate Lanyi, Oleta Williams, Fiona Beyer, Dawn Craig, Barbara Hanratty

**Affiliations:** 1 Population Health Sciences Institute, Newcastle University, Newcastle upon Tyne, UK; 2 School of Health Sciences, University of Manchester, Manchester, UK

**Keywords:** Carers, Older people, Interventions, Outcomes, Evidence gap maps

## Abstract

**Background:**

Unpaid carers of older people, and older unpaid carers, experience a range of adverse outcomes. Supporting carers should therefore be a public health priority. Our understanding of what works to support carers could be enhanced if future evaluations prioritise under-researched interventions and outcomes. To support this, we aimed to: map evidence about interventions to support carers, and the outcomes evaluated; and identify key gaps in current evidence.

**Methods:**

Evidence gap map review methods were used. Searches were carried out in three bibliographic databases for quantitative evaluations of carer interventions published in OECD high-income countries between 2013 and 2023. Interventions were eligible if they supported older carers (50 + years) of any aged recipient, or any aged carers of older people (50 + years).

**Findings:**

205 studies reported across 208 publications were included in the evidence map. The majority evaluated the impact of therapeutic and educational interventions on carer burden and carers’ mental health. Some studies reported evidence about physical exercise interventions and befriending and peer support for carers, but these considered a limited range of outcomes. Few studies evaluated interventions that focused on delivering financial information and advice, pain management, and physical skills training for carers. Evaluations rarely considered the impact of interventions on carers’ physical health, quality of life, and social and financial wellbeing. Very few studies considered whether interventions delivered equitable outcomes.

**Conclusion:**

Evidence on what works best to support carers is extensive but limited in scope. A disproportionate focus on mental health and burden outcomes neglects other important areas where carers may need support. Given the impact of caring on carers’ physical health, financial and social wellbeing, future research could evaluate interventions that aim to support these outcomes. Appraisal of whether interventions deliver equitable outcomes across diverse carer populations is critical.

**Supplementary Information:**

The online version contains supplementary material available at 10.1186/s12877-024-04897-3.

## Background

Across the world, the number of people living with multiple long-term conditions is growing [1, 2]. Trends in disability-free life expectancy also point to an expansion of later life disability in many countries, including the UK [3]. Together, these changes in population health are likely to result in an increased need for care– a scenario that is already predicted for England [4]. 

This increased demand for care presents a critical test for long-term care sectors. Many countries are already facing the challenge of insufficient long-term care funding, and poor availability and quality of services [5]. In England, reductions in funding for adult social care in the past ten years have led to an estimated £6.1 billion deficit [6]. Crucially, the expected growth in need for care in the English population will not be met by the existing supply of paid services [7]. 

These demographic trends point to an urgent need for a comprehensive social care policy in England. In 2021, the adult social care white paper promised reform. However, critics have highlighted a paucity of funds available to galvanise progress, especially in light of the mounting challenges faced by the sector [8]. There have been a number of changes and pauses to policy proposals leading to further delays with current intended reforms [9]. In the absence of a policy that ensures people have equitable access to timely and high-quality formal care, we can expect an unprecedented demand on friends and family to fill the care gap.

Unpaid carers have long been a critical resource in the UK care landscape. However, the current situation means that carers will occupy an even greater role in supporting people living with disability and long-term conditions. Recent analysis suggests that two-thirds of UK carers feel they have no choice about their role [10]. Although some family and friends report caring to be a rewarding experience, the reality for many is that caring can bring adverse consequences– to carers’ health, quality of life, and their social and financial wellbeing [11]. Unpaid care is a clear determinant of health; unpaid carers should therefore be a public health priority [12]. 

### Support for carers: state of current evidence

In the absence of a social care policy for England that ensures people’s needs can be met by existing formal care provision, greater support for carers is imperative. This requires evidence about what approaches work best to support carers. Whilst evaluations of carer interventions are not in short supply, our past work reveals two key failings of this evidence [12]. 

First, some evaluations of carer interventions use inappropriate outcomes as an indicator of success. For example, in studies of carer respite, the outcomes evaluated are predominantly depression and anxiety. However, it is not reasonable that episodic and time-limited respite could feasibly improve long-term mental health outcomes. To identify the value of interventions for carers, evaluations must consider outcomes where meaningful impact can be expected and measured.

Second, systematic review evidence reflects a limited range of interventions for carers. Typically, past systematic reviews have focused on respite and therapeutic supports, such as counselling. Other potential supports and interventions, such as those focusing on supporting physical and financial wellbeing, are largely absent from the synthesised review literature.

In consultation with stakeholders who work across policy and practice to support carers, our work led to the creation of a logic model to depict the range of potential types of interventions that could support carers, and the expected outcomes such interventions could benefit (Table 1) [12]. Each approach may impact one or more of different aspects of carers’ lives, including their physical and/or mental health, quality of life, and their social and financial wellbeing.

**Table 1:** Interventions and outcomes for carers (adapted from Spiers et al., 2021)

Interventions	Outcomes
- Therapeutic talking-based support (e.g. counselling, cognitive behaviour therapy) - Respite - Aids and adaptations for the care recipient that benefit the carer - Financial information and advice - Physical activity and exercise - Physical skills training (e.g. manual handling and transferring skills) - Educational and motivational support - Pain management (targeting pain experienced by the carer, rather than the care recipient) - Befriending and peer support	- Physical health - Mental Health - Carer ‘burden’ - Quality of life - Social wellbeing (e.g. relationships, social participation) - Financial wellbeing

Understanding what works to effectively support carers will likely be a strong feature of the future global health and social care policy research agenda. To support this, we sought to identify and map contemporary international evidence about interventions to support carers, and the outcomes evaluated. This approach offered an opportunity to identify combinations of interventions and outcomes that have been subject to little or no scientific evaluation. Such information may inform policy efforts to support carers by prioritising under-researched yet promising interventions that could be developed, trialled and evaluated.

## Aims of the work

To inform future research about how best to support carers, this work aimed to: map evidence about interventions to support carers, and the outcomes evaluated; and identify key gaps in this evidence.

## Methods

To address the aims of this work, we used evidence gap map methods. This approach supports the visualisation of evidence, including volume, scope and gaps [13]. The methods are reported according to the Preferred Reporting Items for Systematic Reviews and Meta-Analyses [14]. 

### Review criteria

The review criteria are summarised in Table 2. We included evaluations of interventions for any aged carers of older people, and older carers of any aged recipients. Eligible interventions were those that aligned to the categories in Table 1. These interventions are considered relevant to support carers, and were identified through a previous review and stakeholder consultation [12]. We included interventions that targeted carers, or interventions for both care recipient and carer where there was a clear component designed for the carer. Interventions that targeted only outcomes for care recipients were not eligible.

### Search strategy

A targeted search strategy was designed to identify peer-reviewed, published evaluations of interventions for carers. Searches were carried out in three bibliographic databases for the period 2013–2023:

- MEDLINE R (OVID) 1946 to Jan Week 4 2023.
- CINAHL (EBSCO) 1981 to February 2023.
- PsycINFO (OVID) 1806 to January Week 4 2023.

Search terms were adapted for each database. No published filters were used, and searches were not limited by language or publication status. The search strategy applied to Medline is provided in supplementary materials. References were downloaded to, and deduplicated in EndNote (Version 20, Thomson Reuters, New York, USA). All records were then transferred to EPPI-Reviewer software for screening and coding [15]. 

### Screening

Titles and abstracts were screened for relevance. At this stage, 5% of records were screened by all reviewers and discrepancies were discussed to clarify eligibility criteria. The remaining records were screened by single reviewers. The full texts of relevant records were retrieved and assessed against the review criteria, again by single reviewers.

**Table 2:** Review criteria

	Include	Exclude
**Population**	Carers (any age) of people aged 50 + years, and carers aged 50 + years (of any aged care recipient). Study populations without age criteria, but which described carers or care recipients as older or diagnosed with dementia, were eligible.	
**Intervention**	Interventions that comprised one or more of the following components: aids and adaptations, therapeutic support (e.g. counselling, psychotherapy, cognitive behaviour therapy), physical therapy or exercise/activity, respite, pain management, physical skills training, befriending and peer support, financial information and advice, and generic education/advice. Interventions targeted at carers, or dyadic interventions for both care recipient and carer if there is a clear component designed for the carer.	Interventions for care recipients only.
**Comparator**	Any comparator including usual care, or no comparator (i.e. before and after).	
**Outcome**	Any measure of the following: mental health, physical health, carer ‘burden’, financial wellbeing, social wellbeing (including relationships), and quality of life.	
**Study design**	Quantitative evaluative study designs (randomised controlled trials, randomised trials with two intervention arms and no standard care/no intervention arm, non-randomised controlled trials, and before and after studies). Studies published in English from OECD high-income countries; studies published in the last 10 years (2013–2023).	Qualitative evaluations.

Evaluations were eligible if they reported the following outcomes: physical health and/or mental health, carer ‘burden’, quality of life, social wellbeing, and financial wellbeing. These outcomes represent areas where carers may need support, and were informed through our previous work and stakeholder consultation [12]. Although carer ‘burden’ is a contentious concept [16], we opted to include it in this mapping review as it remains a common outcome measure for evaluating carer interventions. To capture all relevant evidence, any measures of the above outcomes were eligible.

We included randomised controlled trials, randomised trials with two intervention arms and no standard care/no intervention arm, non-randomised controlled trials, and before and after studies. Qualitative evaluations were not eligible. Such qualitative study designs typically consider process outcomes, which were not within scope for this mapping review. Studies were eligible if published in English from Organisation for Economic Co-operation and Development (OECD) high-income countries [17]. We used this criterion to identify and map interventions that are most likely to have relevance to the UK policy and population context. To prioritise contemporary evidence, we included studies published in the last 10 years (2013–2023).

### Data extraction

Studies were coded in EPPI-Reviewer to identify key study characteristics: population (carer of older people, older carer, both); intervention type; outcomes evaluated; study design; and, whether analyses considered equity of intervention outcomes by reporting effectiveness across population groups defined by the PROGRESS Plus framework [18]. Where the same study details were reported across two or more publications, we extracted data from the publication with the most detailed information.

### Quality assessment

Quality assessment is not required for an evidence map [19]. However, to identify intervention-outcome combinations with the most robust evidence, we coded studies within EPPI-Reviewer to distinguish by design: randomised + control group, randomised + no control group (two or more intervention arms only), non-randomised + control group, non-randomised + no control group. For this approach, randomised studies with a control group were considered to have the lowest risk of bias. Non-randomised studies without a control group (i.e. pre post-test designs) were considered to have the highest risk of bias.

### Synthesis

Study data were visualised using EPPI mapper software [15]. This method summarises the coverage and volume of evidence across interventions and outcomes, in the form of interactive maps. Map filters distinguished the volume of evidence by study population (carers of older people, older carers, or both), and by study design as an indicator of quality.

## Findings

**Fig. 1:**
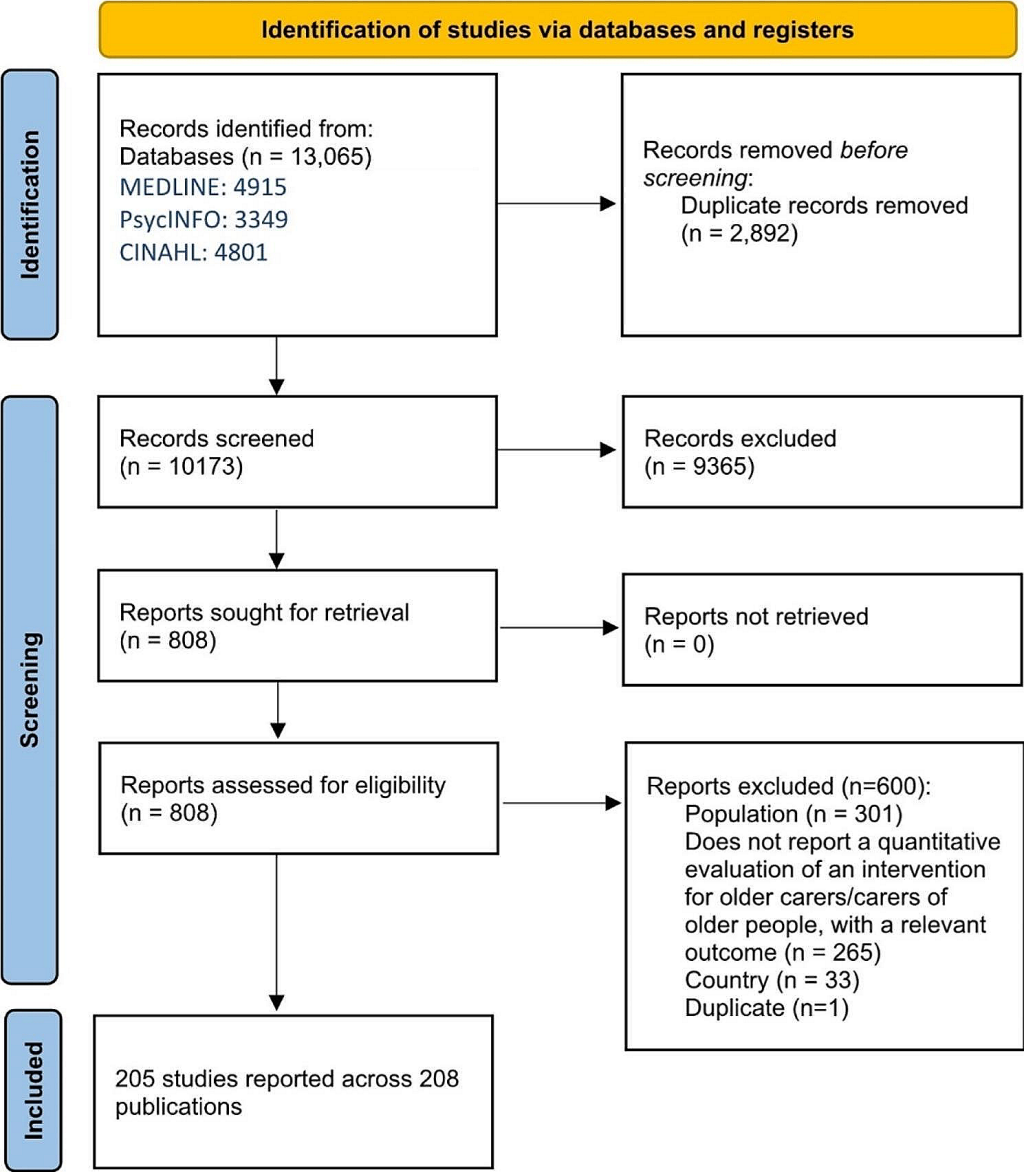
Prisma flow chart

After screening, 205 studies reported across 208 publications were included (Fig. 1; Table 3) [20–227]. For the remainder of this report, we refer to the 205 studies, and *not* the 208 publications.

**Table 3:** Characteristics of included studies (*n* = 205)

Population	
Older carers caring for any-aged care recipient	6
Carers of any adult age caring for older people	157
Older carers (any aged recipient) and carers of older people	42
Interventions*	
Therapeutic	133
Aids and adaptations	17
Physical therapy and activity	22
Pain management	3
Physical skills training (e.g. manual handling and transferring)	7
Financial information and advice	4
Befriending and peer support	25
Respite	6
Education	102
Outcomes*	
Mental health and cognition	172
Physical health	47
Carer burden	105
Financial**	2
Social and relationships	42
Quality of life	60
Cost effectiveness/analysis	9
**Study design**	
Randomised + control group	91
Randomised + no control group (only 2 + treatment arms)	17
Non-randomised and control group	20
Non-randomised and no control group	77
**Do studies report equity in intervention outcomes for carers?**	
Yes	8
No	197

*Numbers not mutually exclusive where interventions included more than one component or multiple outcomes were evaluated; **Financial refers to the carers’ financial circumstances and does not include evaluations of cost effectiveness

The majority of studies evaluated interventions for carers of older people (*n* = 157). Around one fifth (*n* = 42) of studies evaluated interventions for both carers of older people and older carers. Few studies focused only on older carers (*n* = 6). The interventions evaluated typically included more than one component. The most common intervention components were therapeutic (*n* = 133) and educational (*n* = 102). A small number of studies evaluated interventions that focused on pain management for carers (*n* = 3), financial information and advice (*n* = 4), respite (*n* = 6) and physical skills training (*n* = 7). Outcomes evaluated were predominantly mental health and cognition (*n* = 172) and carer burden (*n* = 105). Just two studies evaluated carers’ financial outcomes, which included an assessment of out-of-pocket costs and self-reported financial problems.

A minority of studies (*n* = 8) considered equity. In these studies, the effectiveness of interventions for carers was examined by age categories (*n* = 3), sex (*n* = 2), educational level (*n* = 2), a measure of health status or long-term condition (*n* = 2), employment (*n* = 1), income (*n* = 1), urban versus rural (*n* = 1), area of residence (*n* = 1), ethnicity (*n* = 1), and the level of care recipients’ disability (*n* = 1).

### Evidence gaps

Two evidence gap maps are available at https://tinyurl.com/554t5j49 and https://tinyurl.com/3578ht4t. Both maps show the volume of studies identified by intervention and outcome to highlight concentrations and gaps in evidence. Map A includes a filter for the study populations (carers of older people, older carers, both); map B a filter for study design.

The maps show a clear concentration of studies evaluating the impact of therapeutic and educational interventions on carers’ mental health and burden. By comparison, there are gaps in evidence about the effectiveness of:

- financial information and advice interventions on all outcomes;
- pain management interventions on all outcomes;
- physical skills training on all outcomes.

There was also a smaller amount of evidence about physical activity and therapy interventions, befriending and peer support interventions and aids and adaptations. However, this evidence largely evaluated effectiveness using measures of carer burden and mental health. Less evidence was identified about how these interventions impact on other carer outcomes, including physical health, quality of life, and social wellbeing.

Few studies were identified that evaluated the effectiveness of respite interventions. This is likely to reflect the contemporary time window we used for this work (2013-present), rather than a complete absence of evidence. Our past work identified a large body of evidence about respite for carers published prior to 2013 and which has already been synthesised in two high-quality reviews [228, 229]. 

Evidence map B shows the gaps in evidence by study design. Over half of the studies used randomised designs. Most combinations of interventions and outcomes were represented by evidence from studies using both stronger (randomised) and weaker (non-randomised, pre-post test) designs. Some combinations of interventions and outcomes were represented only by stronger evidence from studies using randomisation. No combinations were represented only by weaker evidence from non-randomised studies. Pre-post test designs were most common for evaluations of therapeutic interventions for carers’ mental health (*n* = 43). However, there was also a similar number of studies using the strongest study design (randomised with control group) for this intervention and outcome combination.

## Discussion

We mapped contemporary evidence on evaluations of carer interventions and associated outcomes. This exercise reveals a strong trend towards the evaluation of therapeutic and educational approaches to supporting carers, prioritising impacts on mental health and burden. The paucity of studies evaluating other types of interventions and outcomes means that we know little about important areas where carers may need support. For example, carers’ physical health may be compromised due to the physical demands of care tasks, or because they neglect their own health when prioritising the wellbeing of the care recipient [230, 231]. Carers also experience adverse financial outcomes, a situation that is deteriorating with the rising cost of living [232–243]. Yet our evidence map suggests that little research in the past ten years has considered what works best to support carers’ physical and financial wellbeing. Other key gaps in evidence include consideration of a range of interventions on carers’ social wellbeing and quality of life.

Most combinations of interventions and outcomes were represented by evidence from both stronger (randomised) and weaker (non-randomised, pre-post test) designs. No combination of interventions and outcomes were represented only by pre-post test designs, which we consider to be the weakest. This suggests that the primary limitation of this evidence base is the poor representation of a range of interventions and outcomes, as opposed to the strength of study designs to answer questions of effectiveness.

The gaps in evidence that we identified may reflect two scenarios in the applied health sciences. The first scenario is that an intervention may have been subject to evaluation in the period preceding the time window for the present work (2013–2023), and is no longer a focus of contemporary research. This may be the case for respite interventions, which have been comprehensively evaluated in the past [12]. Previous reviews have reported inconsistent evidence about the effectiveness of respite for carers [228, 229]. The small number of evaluations on respite identified here may reflect this pattern of earlier evidence, as well as changing research priorities and evolving approaches to supporting carers.

The second scenario is that an intervention has not yet been subject to extensive (or indeed any) evaluative study. This may be due to several reasons, including funding, feasibility considerations (such as populations being too small for rigorous evaluation), or that the intervention is new and still being developed.

Very few evaluations reported data to ascertain whether interventions produced equitable outcomes. Future studies should address this gap by exploring the extent to which any observable benefits of interventions are experienced across diverse groups of carers. This is important because outcomes for carers are socially patterned. For example, carers who are female, single, in poor health and who experience socioeconomic disadvantage are especially vulnerable to the adverse financial consequences of caring [237, 239, 240, 243, 244]. 

A key strength of this work is our consideration of evidence across a range of interventions and outcomes. This has enabled us to pinpoint combinations of interventions and outcomes that lack evidence, which can inform future directions in research to support carers. Comprehensive searches and robust, transparent review methods underpin the rigour of this work. Our approach did not consider grey literature, which may include local evaluations and audits that are not published. However, as researchers in this field, our expectation is that there are unlikely to be any high quality, quantitative evaluations published in non-peer reviewed sources.

### Implications for policy and future research

Supporting carers is increasingly recognised as essential to the wider care system. The 2014 Care Act granted carers the right to an assessment of their needs and the right to have eligible needs met [245]. Alongside these Care Act duties, the recent passing of the Carers’ Leave Act [246] signals greater recognition of the role of carers, and their right to be supported, rather than marginalised, members of society. Finding ways to support carers is critical. Future research about what works best to support carers should consider interventions that can address outcomes beyond burden and mental health alone. Evaluating a broader range of interventions, including those based on physical activity, financial information and advice, befriending and peer support, physical skills training and pain management, would enhance the breadth of current evidence.

Third sector organisations play a key role in advocating for carers, and are likely to drive innovative approaches to delivering inclusive support. However, evaluations of these approaches can be small scale and with limited funding. Enhancing partnerships and opportunities for co-production between these organisations and academic sectors could enhance evidence about what works to support carers.

The dominance of burden and mental health outcomes may reflect a medical model approach to considering support for carers. Shifting to a more holistic social model may help to address some of the gaps in social and financial outcomes that we observed, whilst also attending to issues of equity and inclusion of diverse carer populations.

Finally, whilst qualitative process evaluations were not within the scope of this map, such methods nonetheless provide critical insight into how (and under what conditions) interventions meet carers’ needs. Thus, going forward, any evaluation of support for carers should integrate quantitative and qualitative designs to maximise the evidence base available for policy makers.

## Conclusion

Contemporary evidence about what works best to support carers is vast in quantity but limited in scope. Future commissioning of research for carers may benefit from seeking evaluations of interventions with little or no evidence. Consideration of how such interventions impact a range of outcomes for carers, including their financial wellbeing and physical health, is critical. Appraisal of whether interventions deliver equitable benefits across diverse groups of carers will address an important evidence gap.

### Electronic supplementary material

Below is the link to the electronic supplementary material.

Supplementary Material 1

## Data Availability

Data sharing is not applicable to this article as no datasets were generated or analysed during the current study.
