# Supplementary material for: What works to support carers of older people and older carers? an international evidence map of interventions and outcomes
Source: BMC Geriatr. 2024 Mar 29;24:301. doi: 10.1186/s12877-024-04897-3 (PMC10979610; doi:10.1186/s12877-024-04897-3)
Supplement: Supplementary file 1 — Supplementary Material 1 [file 12877_2024_4897_MOESM1_ESM.docx]

Supplementary materials

Appendices:

**MEDLINE search strategy:**

Ovid MEDLINE(R) <1946 to January Week 4 2023>

| **#** | **Query** | **Results from 3 Feb 2023** |
| --- | --- | --- |
| 1 | *Caregivers/ | 31,443 |
| 2 | older care*.ti. | 117 |
| 3 | (caregiver* or "care giver*").ti. | 21,189 |
| 4 | (carer or carers).ti. | 3,911 |
| 5 | or/1-4 | 36,786 |
| 6 | psychotherapy/ or animal assisted therapy/ or art therapy/ or emotion-focused therapy/ or interpersonal psychotherapy/ or music therapy/ or narrative therapy/ or person-centered psychotherapy/ or play therapy/ | 65,071 |
| 7 | exp behavior therapy/ | 87,511 |
| 8 | exp Counseling/ | 48,335 |
| 9 | counsel*.tw,kw. | 112,814 |
| 10 | psychotherap*.kw,tw. | 46,566 |
| 11 | therap* support.kw,tw. | 721 |
| 12 | ((mindfulness or "mindfulness-based therap*") adj practi?e).kw,tw. | 366 |
| 13 | or/6-12 | 293,729 |
| 14 | Physical Therapy Modalities/ | 40,287 |
| 15 | Exercise/ | 137,816 |
| 16 | exp Exercise Therapy/ | 61,853 |
| 17 | Pain Management/ | 40,176 |
| 18 | physical therap*.kw,tw. | 25,910 |
| 19 | physical activit*.kw,tw. | 124,952 |
| 20 | (manual adj (mov* or handling or lifting)).kw,tw. | 1,011 |
| 21 | pain manag*.kw,tw. | 26,708 |
| 22 | or/14-21 | 358,869 |
| 23 | (assistive adj (technolog* or product* or device*)).kw,tw. | 4,666 |
| 24 | (home adj (adaptation* or modification*)).kw,tw. | 314 |
| 25 | or/23-24 | 4,936 |
| 26 | Social support/ or Psychosocial Support Systems/ | 78,876 |
| 27 | ((financ* or social or peer) adj support).kw,tw. | 51,260 |
| 28 | befriend*.kw,tw. | 362 |
| 29 | or/26-28 | 104,465 |
| 30 | Respite Care/ | 1,088 |
| 31 | respite.kw,tw. | 1,866 |
| 32 | or/30-31 | 2,334 |
| 33 | *Caregivers/ed [Education] | 1,595 |
| 34 | 5 and (13 or 22 or 25 or 29 or 32) | 9,149 |
| 35 | 33 or 34 | 10,355 |
| 36 | limit 34 to yr="2013 - 2023" | 4,915 |
